# Predictive utility of the impedance drop on AF recurrence using digital intraprocedural data linked to electronic health record data

**DOI:** 10.1016/j.hroo.2024.01.006

**Published:** 2024-02-02

**Authors:** Paul Coplan, Amit Doshi, Mingkai Peng, Yariv Amos, Mati Amit, Don Yungher, Rahul Khanna, Liat Tsoref

**Affiliations:** ∗ MedTech Epidemiology and Real-World Data Sciences, Office of the Chief Medical Officer, Johnson & Johnson, New Brunswick, New Jersey; † Perelman School of Medicine at the University of Pennsylvania, Philadelphia, Pennsylvania; ‡ Mercy Hospital, St. Louis, Missouri; § Biosense Webster LTD, Haifa Technology Center, Israel

**Keywords:** Atrial fibrillation, Impedance drop, Radiofrequency catheter ablation, CARTONET, Linkage

## Abstract

**Background:**

Local impedance drop in cardiac tissue during catheter ablation may be a valuable measure to guide atrial fibrillation (AF) ablation procedures for greater effectiveness.

**Objective:**

The study sought to assess whether local impedance drop during catheter ablation to treat AF predicts 1-year AF recurrence and what threshold of impedance drop is most predictive.

**Methods:**

We identified patients with AF undergoing catheter ablation in the Mercy healthcare system. We downloaded AF ablation procedural data recorded by the CARTO system from a cloud-based analytical tool (CARTONET) and linked them to individual patient electronic health records. Average impedance drops in anatomical region of right and left pulmonary veins were calculated. Effectiveness was measured by a composite outcome of repeat ablation, AF rehospitalization, direct current cardioversion, or initialization of a new antiarrhythmic drug post–blanking period. The association between impedance drop and 1-year AF recurrence was assessed by logistic regression adjusting for demographics, clinical, and ablation characteristics. Bootstrapping was used to determine the most predictive threshold for impedance drop based on the Youden index.

**Results:**

Among 242 patients, 23.6% (n = 57) experienced 1-year AF recurrence. Patients in the lower third vs upper third of average impedance drop had a 5.9-fold (95% confidence interval [CI] 1.81–21.8) higher risk of recurrence (37.0% vs 12.5%). The threshold of 7.2 Ω (95% CI 5.75–7.7 Ω) impedance drop best predicted AF recurrence, with sensitivity of 0.73 and positive predictive value of 0.33. Patients with impedance drop ≤7.2 Ω had 3.5-fold (95% CI 1.39–9.50) higher risk of recurrence than patients with impedance drop >7.2 Ω, and there was no statistical difference in adverse events between the 2 groups of patients. Sensitivity analysis on right and left wide antral circumferential ablation impedance drop was consistent.

**Conclusion:**

Average impedance drop is a strong predictor of clinical success in reducing AF recurrence but as a single criterion for predicting recurrence only reached 73% sensitivity and 33% positive predictive value.

**Key Findings**

▪ Low impedance drop during cardiac ablation procedures to treat atrial fibrillation (AF) is associated with a high risk of AF recurrence.
▪ Bootstrap analysis identified the threshold for impedance drop to be 7.2 Ω, with a median sensitivity of 0.73 and specificity of 0.54 to predict AF recurrence.
▪ Linkage between ablation procedural data and patients’ electronic health data may be used to improve patient outcomes at the population level.

## Introduction

Pulmonary vein (PV) isolation using radiofrequency (RF) catheter ablation is the predominant approach to ablation of atrial fibrillation (AF).^1^ However, AF recurrence after catheter ablation remains problematic. In the Catheter ABlation vs ANtiarrhythmic Drug Therapy for Atrial Fibrillation (CABANA) trial, 12.6% of patients experienced a recurrence of symptomatic AF and 36.4% of patients had a first recurrence of any AF within 12 months postablation.^2^ The goal of the RF ablation is to create lesions to stop conduct of aberrant electrical signals that induce atrial arrhythmia by means of electrical-current resistive heating. Durable lesions with sufficient size and depth are critical for the success of ablation procedure. Contact force–sensing catheters allow for a delivery of ablation at specific power, contact force, and duration.^3^ Also, generator impedance drop is routinely monitored in clinical practice to assess RF therapy delivery.^4^ Analyzing ablation procedural data related to lesion formation could allow us to optimize the process of RF delivery and improve patients’ outcome.^5^

Impedance drop occurs during the RF ablation process and depends on tissues’ thickness and anatomical locations. Previous studies on impedance drop have been focusing on how impedance drops at the lesion level impact the formation of lesions and PV segment isolation. For example, the Electrical Coupling Information From The Rhythmia HDx System And DirectSense Technology In Subjects With Paroxysmal Atrial Fibrillation (LOCALIZE) trial found local impedance drop was predictive of PV isolation segment conduction block.^4^ Both absolute and percentage change of impedance drop were associated with successful lesions in a study of 1633 RF applications from 23 patients.^6^ Meanwhile, various threshold values have been reported in the literature. For example, 21 of 25 patients with paroxysmal AF remained free of recurrent AF after the mean follow-up of 431 ± 87 days by ensuring at least 5 Ω of impedance drop in the first 10 seconds of ablation.^7^ Recovered PV conduction occurs predominantly in regions where adjacent ablation applications result in impedance decrease of <10 Ω.^8^

In this study, an epidemiological approach compares average impedance drop at left and right wide antral circumferential ablation (WACA) with clinical outcomes defined by diagnosis and procedure codes using real-world data. Procedural data collected during ablation were linked with patients’ electronic health record (EHR) data. The study was built upon a test case funded by the National Evaluation System for Health Technology, a program to foster real-world evidence use for regulatory decisions about medical devices.^9^^,^^10^ The data quality of the Mercy health EHR system for studies of cardiac ablation catheters to treat AF were assessed and quantified.^11^^,^^12^ The aim was to assess 2 points: (1) Does average impedance drop at right and left WACA predict effectiveness in reducing 1-year AF recurrence? and (2) If yes, what threshold of impedance drop best predicts AF recurrence?

## Methods

### Data sources

This study used the linked EHR data collected using Epic software (Epic Systems, Verona, WI) with procedural data collected during RF catheter ablation procedure for AF patients in Mercy healthcare system. All CARTO 3 system (Biosense Webster, Irvine, CA) data were scrubbed of protected health information and transmitted via the Siemens teamplay platform (Siemens, Malvern, PA) to the Microsoft Azure cloud (Microsoft, Redmond, WA). CARTONET (Biosense Webster) is a cloud-based storage and analysis software for CARTO data processing and aggregation. Procedure data were anonymized by CARTONET, with lesion locations and corresponding impedance reductions derived from the Visitag module. The centroid of all catheter locations during each RF determined the location of lesions in the analytical software, which were automatically assigned to regions of the left WACA, right WACA, and additional linear lesions with correction by a human observer as necessary. Detailed information on how the CARTONET collect, store, and analyze the data has been previously published.^5^

### Study population

Mercy healthcare system operates in 4 states in the Midwest United States, with over 40 hospitals, 12 outpatient surgery centers and 35 urgent care sites, and provides care to approximately 4.2 million patients. Patients (19 years of age and older) with AF (International Classification of Diseases–Tenth Revision–Clinical Modification diagnostic code I48.X) undergoing catheter ablation (identified using International Classification of Diseases–Tenth Revision procedure codes [ICD-10-PCS] [02563ZZ, 02573ZZ, 025K3ZZ, 025L3ZZ, 02583ZZ, 02553ZZ, 025M3ZZ, 025S3ZZ, 025T3ZZ]/Current Procedural Terminology [CPT] codes [93656]) using either the THERMOCOOL SMARTTOUCH catheter (Biosense Webster) or THERMOCOOL SMARTTOUCH SF ablation catheter (Biosense Webster) in an inpatient or outpatient setting between January 1, 2016, and December 31, 2018, were included. The first occurrence of cardiac ablation procedure was considered as index ablation. Patients who were continuously enrolled in Mercy health for at least 12 months pre–index and 12 months post–index period were included. Patients with catheter ablation procedure for AF within 6 months prior to the index date were excluded.

Procedural data including impedance drop were downloaded from CARTONET for all ablation sites for each patient and were linked with Mercy health data using the unique patient identifier and CARTONET identifier. The unique linkage identifier between EHR data and intraprocedural machine data was extracted by a nurse from Mercy trained in data extraction. Impedance drop was aggregated in 2 steps. First, average impedance drops for right and left WACAs were calculated. WACA is defined as circumferential isolation performed ≥1.5-cm away from the PV ostium identified by angiography or 3-dimensional electroanatomic reconstruction. Second, the average impedance drop of WACA is the average of impedance drop in right and left WACA area calculated at the first step. Assignment of individual lesions to the right and left WACAs was initially performed automatically through machine learning tools built into CARTONET and subsequently reviewed manually by trained CARTONET users. Average stability (mm), average force (g), and maximum power (W) at right and left WACAs were calculated for each patient. Indicators were created on whether ablation was conducted at right/left carina and outside of right and left PVs.

Patient demographics including age and sex, and clinical characteristics including atrial flutter, AF type, sleep apnea, CHA_2_DS_2_-VASc (congestive heart failure, hypertension, age ≥75 years, diabetes mellitus, prior stroke or transient ischemic attack or thromboembolism, vascular disease, age 65–74 years, sex category) score, Elixhauser comorbidity index, and use of antiarrhythmic drug prior to ablation were extracted and calculated from Mercy EHR data.

### Study outcome

The outcome of the study was 1-year AF recurrence defined as a composite of (1) repeat ablation using ICD-10-PCS codes or CPT codes with a diagnosis code of AF, (2) hospitalization with the principal diagnosis of AF, (3) direct current cardioversions using the ICD-10-PCS code 5A2204Z and CPT codes 92960 and 92961, or (4) initialization of a new antiarrhythmic drug (including amiodarone, disopyramide, dronedarone, sotalol, propafenone, flecainide, dofetilide, quinidine, and ibutilide; post–blanking period) using national drug codes or text searches. The composite outcome was similar to the clinical effectiveness ends point used in the clinical trial, except without the atrial arrhythmia recurrence by ambulatory monitor or electrocardiography (ECG).^13^ There were no recordings from ambulatory monitor or ECG included in this study because these values are not routinely captured in EHR databases.

To understand the availability of longitudinal data for outcome ascertainment, we determined the duration of follow-up through patient encounters with the Mercy Health system at 1 year. Only encounters between the inpatient and the healthcare system within 1 year postablation of interest were included. Follow-up was defined as an encounter identified using an algorithm of in-person contact that included both face-to-face visits and remote contact, such as telephone visits, with any representative at the given health system. Using this approach we identified that over 66% of ablated AF patients had a documented healthcare interaction with the Mercy health system after 1 year following their initial AF event, and therefore were not lost to follow-up.^11^

The composite endpoints of adverse event was defined as 1 of 14 safety events: death, acute myocardial infarction, acute stroke, transient ischemic attack, thromboembolism, diaphragmatic paralysis, pneumothorax, heart block, pulmonary edema, pericarditis, major vascular access complication, or bleeding that required transfusion within 7 days after the procedure; and cardiac perforation or tamponade, PV stenosis, or atrioesophageal fistula within 30 days using ICD-10 diagnosis/procedure codes or CPT codes.^14^

### Statistical analysis

Baseline and ablation characteristics of the study population were described using proportions for categorical variables and mean ± SD for continuous variables. Differences between patients with and without AF recurrence outcome were tested using the chi-square test for categorical variables and 1-way analysis of variance test or *t* test for continuous variables.

Multivariable logistic regression was used to estimate the association of impedance drop with the composite outcome. Covariates included patients’ age and sex, AF type, sleep apnea, atrial flutter, history of antiarrhythmic drug before ablation, CHA_2_DS_2_-VASc score, Elixhauser comorbidity index, average WACA stability, ablation index, contact force, power, indicators of ablation at carina and outside of right/left PVs, left atrium dilation (defined as ratio of posterior length divided by posterior height), total number of ablation lesions, and total ablation duration. Results are presented as odds ratios (ORs) with 95% confidence intervals (CIs). The main analysis evaluated the average of left and right WACA impedance drops. Average impedance drops were categorized into tertiles. Sensitivity analyses were performed using the right and left WACA impedance drop groups.

A threshold for average impedance drop was chosen to identify patients with high risk of 1-year AF recurrence. Bootstrap analysis with 1000 resamplings with replacement was used to search for the threshold value by maximizing the Youden index using the curpointr R package.^15^ Youden index is equal to the sum of sensitivity and specificity minus 1, which balances both sensitivity and specificity.^16^ For each resampling, the threshold was estimated from fit via generalized additive model with smooth terms. Sensitivity, specificity, positive predictive value, negative predictive value, and area under the receiver-operating characteristic curve were presented.

All tests were 2-sided, and a *P* value ≤.05 was considered significant. All analyses were performed using R version: 4.3.0 (R Foundation for Statistical Computing, Vienna, Austria).

## Results

**Figure 1:**
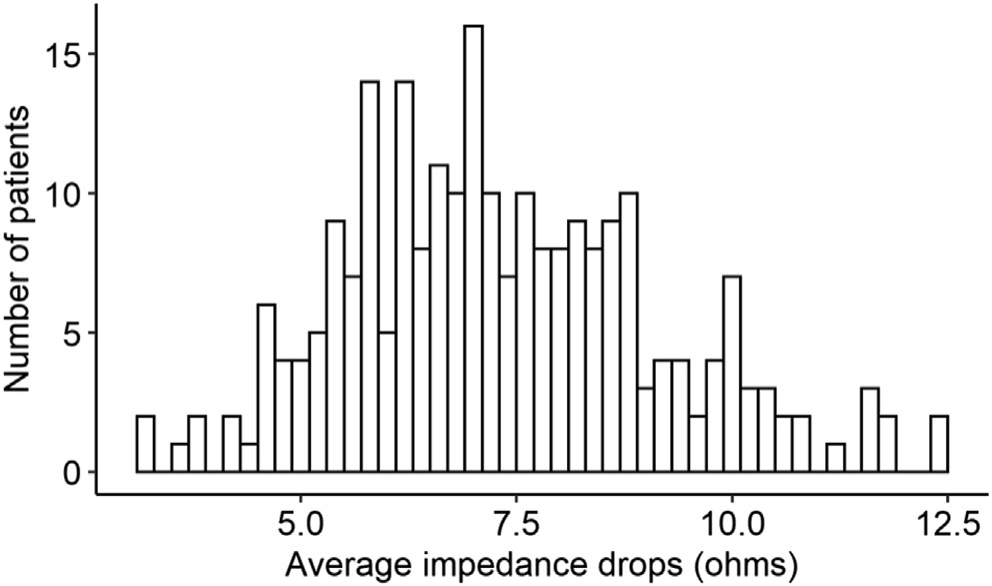
Distribution of average impedance drops.

There were 242 patients in this study. The average age of the sample was 63.4 years, with 68.2% being male (Table 1); 23.5% experienced 1-year AF recurrence postablation. Patients with 1-year AF recurrence were more likely to have persistent AF, small impedance drop in both right and left WACAs, and more ablation lesions compared with patients without 1-year AF recurrence. There was no difference in age, sex, status of sleep apnea, Elixhauser comorbidity index, CHA_2_DS_2_-VASc score, the number of right WACA ablation sites, other ablation parameters among patients with and without 1-year AF recurrence. As shown on Figure 1, the average impedance drops ranged from 3.2 to 12.5 Ω, with a median of 7.1 (interquartile range [IQR] 6.05–8.54). Only 9.5% of patients had the average impedance drop over 10 Ω.

**Table 1:** Baseline and ablation characteristics

	Overall (n = 242)	1-y AF recurrence		*P* value
		No (n = 185)	Yes (n = 57)	
Age, y	63.4 ± 10.0	62.9 ± 9.9	65.0 ± 10.3	.151
Male	165 (68.2)	122 (65.9)	43 (75.4)	.237
AF type				
Paroxysmal AF	111 (46.1)	98 (53.0)	13 (23.2)	<.001
Persistent AF	130 (53.9)	87 (47.0)	43 (76.8)	
Sleep apnea	74 (30.6)	54 (29.2)	20 (35.1)	.496
Atrial flutter	15 (6.2)	14 (7.6)	1 (1.8)	.201
CHA_2_DS_2_-VASc score				
0	42 (17.4)	36 (19.5)	6 (10.5)	.103
1–2	115 (47.5)	90 (48.6)	25 (43.9)	
≥3	85 (35.1)	59 (31.9)	26 (45.6)	
Elixhauser comorbidity index				
0	22 (9.1)	18 (9.7)	4 (7.0)	.199
1–2	88 (36.4)	72 (38.9)	16 (28.1)	
≥ 3	132 (54.5)	95 (51.4)	37 (64.9)	
Average impedance drop				
3.2–6.45 Ω	81 (33.5)	51 (27.6)	30 (52.6)	.001
6.45–8 Ω	81 (33.5)	64 (34.6)	17 (29.8)	
8–12.5 Ω	80 (33.1)	70 (37.8)	10 (17.5)	
Left ventricular ejection fraction				.448
<50% (reduced)	22 (9.1)	16 (8.6)	6 (10.5)	
≥50%	158 (65.3)	118 (63.8)	40 (70.2)	
Missing	62 (25.6)	51 (27.6)	11 (19.3)	
Carina ablation	167 (69.0)	121 (65.4)	46 (80.7)	.043
Ablation outside right and left PV	207 (85.5)	156 (84.3)	51 (89.5)	.453
Antiarrhythmic drug use before ablation	149 (79.3)	110 (82.7)	39 (70.9)	.106
Impedance drop, Ω				
Right WACA	7.1 ± 2.1	7.3 ± 2.1	6.1 ± 1.8	<.001
Left WACA	7.7 ± 2.0	7.9 ± 2.0	7.0 ± 2.0	.003
Ablation sites				
Total	173.6 ± 90.2	164.9 ± 81.8	202 ± 109.2	.006
Right WACA	69.8 ± 35.2	69.4 ± 34.7	71.2 ± 37.0	.728
Left WACA	63.3 ± 40.6	58.9 ± 34.6	77.3 ± 53.9	.003
Ablation index				
Average WACA	367 ± 52.3	369 ± 48	360 ± 63.5	.262
Right WACA	377 ± 58.1	380 ± 54.9	369 ± 67.4	.21
Left WACA	358 ± 52.1	359 ± 48.6	352 ± 62.5	.394
Stability, mm				
Average WACA	1.61 ± 0.55	1.61 ± 0.56	1.59 ± 0.52	.863
Right WACA	1.60 ± 0.57	1.61 ± 0.58	1.58 ± 0.54	.709
Left WACA	1.61 ± 0.57	1.61 ± 0.58	1.61 ± 0.53	.966
Contact force, g				
Average WACA	14.5 ± 4.8	14.8 ± 4.7	13.5 ± 4.9	.078
Right WACA	15.4 ± 5.5	15.8 ± 5.5	14.2 ± 5.4	.056
Left WACA	13.6 ± 4.6	13.8 ± 4.6	12.9 ± 4.7	.172
Power, W				
Average WACA	31.0 ± 4.0	31.0 ± 3.2	30.9 ± 6.0	.806
Right WACA	30.6 ± 4.2	30.8 ± 3.3	30.1 ± 6.3	.285
Left WACA	31.4 ± 5.5	31.3 ± 5.2	31.6 ± 6.4	.648
Total ablation duration (mins)	54.6 ± 23.9	51.9 ± 22.1	63.3 ± 27.5	.001

Values are mean ± SD or n (%).

AF = atrial fibrillation; CHA_2_DS_2_-VASc = congestive heart failure, hypertension, age ≥75 years, diabetes mellitus, prior stroke or transient ischemic attack or thromboembolism, vascular disease, age 65–74 years, sex category; PV = pulmonary vein; WACA = wide antral circumferential ablation.

**Figure 2:**
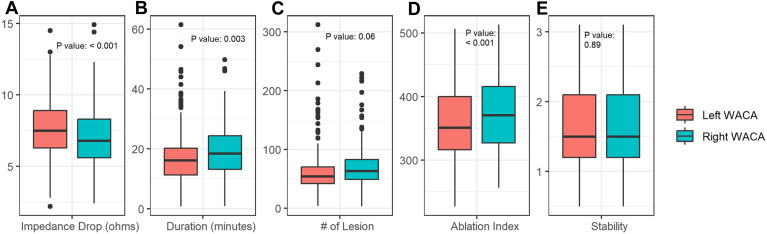
Comparison of digital ablation parameters measured by the CARTO navigation system between right and left atrial wide antral circumferential ablation (WACA). (A) impedance drop, (B) duration, (C) # of lesions, (D) ablation index, (E) stability.

In total, there were 57 patients with 1-year AF recurrence post–index ablation. As shown in Table 2, there were 30 events among 81 (37.0%) patients with impedance drop ≤6.47 Ω (tertile 1 [ie, one-third of patients with the lowest rank of impedance drop]) compared with 10 events among 80 (12.5%) patients with impedance drop >8.05 Ω (tertile 3). Results from regression analysis showed that patients with impedance drop ≤6.47 Ω were 5.9 times more likely (95% CI 1.81–21.8) to have 1-year AF recurrence compared with patients with impedance drop >8.05 Ω when averaged across right and left WACAs. A sensitivity analysis with regression models for right WACA (OR 3.8, 95% CI 1.23–13.0) and left WACA (OR 4.5, 95% CI 1.57–13.5) impedance were consistent. As shown in Figure 2, right WACAs had on average slightly lower impedance drop than left WACAs despite high ablation index values and longer ablation duration for right WACA.

**Table 2:** 1-year AF recurrence composite outcomes for patients

	Patients	Events	Adjusted OR (95% CI)∗
Average impedance drop			
High: >8.05 Ω	80	10 (12.5)	Reference
Medium: 6.47–8.05 Ω	81	17 (21.0)	1.80 (0.57–5.98)
Low: ≤6.47 Ω	81	30 (37.0)	5.91 (1.81–21.8)
Right WACA impedance drops∗			
High: >8.05 Ω	74	10 (13.5)	Reference
Medium: 6.47–8.05 Ω	61	6 (9.8)	0.44 (0.09–1.79)
Low: ≤6.47 Ω	107	41 (38.3)	3.80 (1.23–13.0)
Left WACA impedance drop∗			
High: >8.05 Ω	100	14 (14.0)	Reference
Medium: 6.47–8.05 Ω	75	18 (24.0)	1.50 (0.57–3.98)
Low: ≤6.47 Ω	67	25 (37.3%)	4.48 (1.57–13.5)

Values are n or n (%), unless otherwise indicated. The composite outcome includes AF hospitalization, repeat ablation, direct current cardioversion, and initialization of a new antiarrhythmic drug post–blanking period.

AF = atrial fibrillation; CI = confidence interval; OR = odds ratio; WACA = wide antral circumferential ablation.

∗ The full results of multivariable are presented in the Supplemental Tables 1–3.

**Figure 3:**
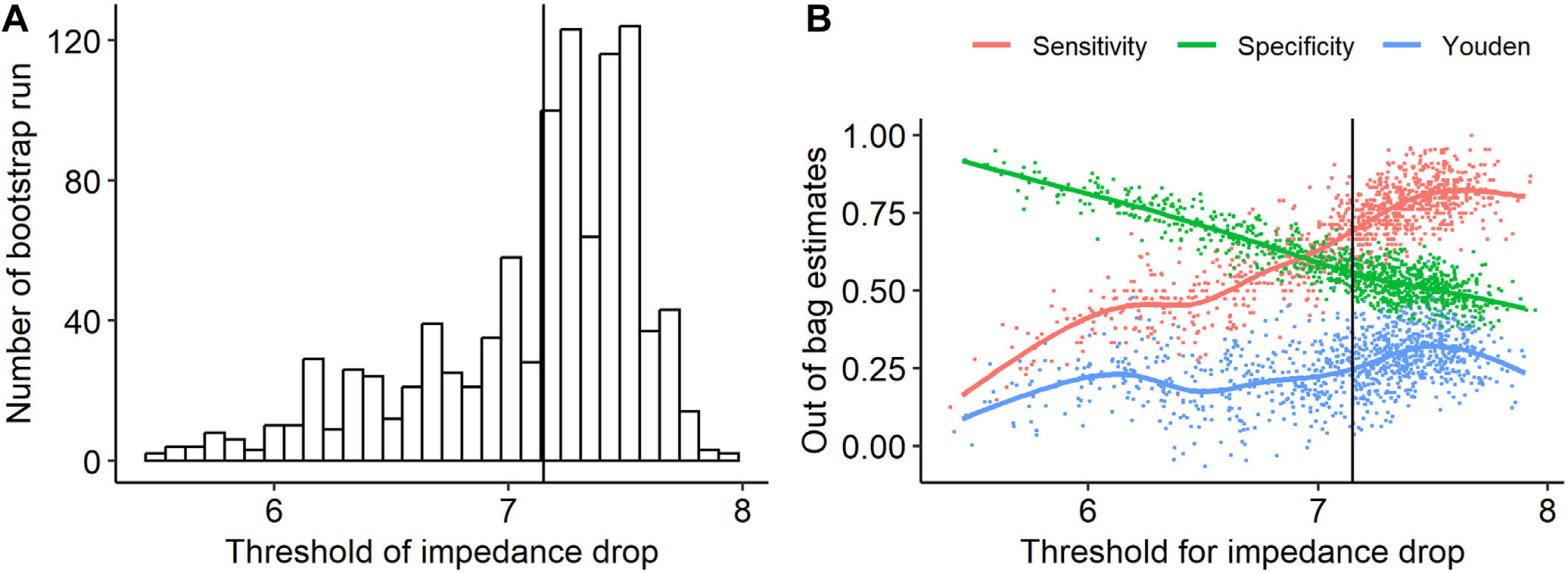
Results from using the Youden index and bootstrapping 1000 samples for selecting the threshold for impedance drop that best predicts 1-year atrial fibrillation recurrence: (A) distribution of threshold of impedance drop (vertical line shows the median value); (B) association between sensitivity, specificity, and Youden index (sensitivity + specificity – 1) and impedance drop threshold values.

Using the bootstrap method, the mean threshold for impedance drop that best differentiate the risk of AF recurrence based on the Youden index was 7.2 Ω (95% CI 5.75–7.65 Ω). Figure 3A shows the distribution of threshold values from the bootstrap steps. Threshold values ranged from 5 to 8, with most values between 7 and 8. Figure 3B shows the relationship between the threshold of impedance drop and out-of-bag estimates of sensitivity, specificity, and Youden index that were calculated from the observations that were not used in the estimation of threshold in each resampling process. Sensitivity increased with the increase of threshold, while specificity decreased with the increase of threshold. The Youden index balanced both sensitivity and specificity when selecting the threshold. The median out-of-bag sensitivity and specificity were 0.73 (IQR 0.56–0.81) and 0.56 (IQR 0.50–0.64), respectively. In other words, 73% of patents with AF recurrence had the impedance drop <7.2 Ω. The median and IQR of out-of-bag positive predictive value and negative predictive value were 0.33 (95% CI 0.30–0.37) and 0.86 (95% CI 0.82–0.90), respectively (Supplemental Figure 1). Using the bootstrap method and Youden index, the threshold was 6.4 Ω for right WACA and 7.3 Ω for left WACA.

Based on the mean threshold of 7.2 Ω selected from bootstrap analysis (Table 3), there were 42 (34.1%) events from 123 patients with average impedance drop ≤7.2 Ω compared with 15 (12.6%) events among 119 patients with impedance drop >7.2 Ω. Results from regression analysis showed that patients with impedance drop ≤7.2 Ω were 3.5 times more likely (95% CI 1.39–9.50) to have 1-year AF recurrence compared with patients with impedance drop >7.2 Ω. The adjusted OR was 4.32 (95% CI 1.57–13.2) for right WACA impedance drop and 2.97 (95% CI 1.27–7.27) for left WACA impedance drop. Six of 7 patients with adverse events had an impedance drop ≤7.2 Ω, and there was no statistically significant difference in adverse event rates between patients with an impedance drop of ≤7.2 Ω and >7.2 Ω.

**Table 3:** 1-year AF recurrence composite outcome for patients with impedance drop above or below the best-predicting threshold

	Patients	Events	Adjusted OR (95% CI)∗
Average impedance drop			
>7.2 Ω	119	15 (12.6)	Reference
≤7.2 Ω	123	42 (34.1)	3.51 (1.39–9.50)
Right WACA impedance drop			
>7.2 Ω	104	12 (11.5)	Reference
≤7.2 Ω	138	45 (32.6)	4.32 (1.57–13.2)
Left WACA impedance drop			
>7.2 Ω	139	24 (17.3)	Reference
≤7.2 Ω	103	33 (32.0)	2.97 (1.27–7.27)

The composite outcome includes AF hospitalization, repeat ablation, direct current cardioversion, or initialization of new antiarrhythmic drugs post–blanking period.

AF = atrial fibrillation; CI = confidence interval; OR = odds ratio; WACA = wide antral circumferential ablation.

∗ A full list of ORs for all the variables in multivariable analysis can be found in Supplemental Tables 4–6.

## Discussion

Cardiac ablation is increasingly becoming a primary modality of treatment for AF.^17^ Better assessment of procedural parameters associated with AF recurrence postablation could optimize ablation strategies and improve patient outcomes. Our study attempts to link ablation procedural digital data with EHR data at a patient level to better understand the association between impedance drop at the anatomical site level and AF recurrence extracted from real-world data through an epidemiological study. Our study showed that a lower impedance drop in WACA was associated with a higher risk of 1-year AF recurrence. We also assessed the threshold of impedance drop that best predicts 1-year AF recurrence, balancing sensitivity and specificity through the Youden index, and found this value to be 7.2 Ω. Preclinical studies have shown correlations between impedance drop and lesion surface area, maximum depth, and lesion volume,^18^ which support our study findings. Our analysis shows an approximately 3.5-fold difference in AF recurrence rates in AF procedures with larger vs smaller impedance drops.

Procedural data generated for each lesion ablation provide valuable information to improve durable lesion formation.^19^ Our study showed that impedance drop is predictive of AF recurrence. The results were consistently for both right and left WACA impedance, although the impedance drop was lower for the right WACA on average than the left WACA. In this study, we used average impedance drop, as it is available from the procedural data summary in CARTO data and increased the sample size for this study. Use of minimal impedance drop in the anatomical region or integration of impedance drop with time may better predict the clinical outcome.^20^

Technological tools like cloud storage provide a novel and feasible way to store very large ablation procedural data and run machine learning models on such stored data on the cloud, and thereby enhance the use of these data for research, qualitive improvement, and reporting.^5^ Linkage with real-world data would allow researchers to create large sample sizes to study the association of procedural parameters with AF recurrence in real-world practice and at the population level. Our study is the first that links ablation procedural data with patients’ EHR data and demonstrates the potential usage of data to optimize the RF workflow systematically using a large study population.

We used the bootstrap method to maximize the Youden index to search for the threshold for impedance drop that best predicts AF recurrence. Out-of-bag performance and variation for the threshold value were evaluated. Thresholds of the impedance drop were consistent across different resamplings. The optimized Youden index is equivalent to the maximum vertical distance between the receiver operating curve and the diagonal line. We also evaluated the threshold by maximizing the diagnostic OR and the product of sensitivity and specificity.^21^^,^^22^ The results from the other 2 metrics were consistent with the result from Youden index. However, the sensitivity and specificity were approximately 0.73 and 0.56, respectively, using the threshold of 7.2 Ω. Although the threshold of 7.2 Ω might have clinical utility as a prospective target during RF energy delivery, this may be limited by the relatively low sensitivity and specificity. Around 70% of patients with average impedance drop <7.2 Ω would not experience AF recurrence due to low positive predictive value. Sensitivities of 0.82 and 0.89 could be achieved by increasing the threshold to 8.0 and 9.0 Ω, respectively, while specificity would drop to 0.39 and 0.28, potentially increasing overablation and concomitant risk of adverse events. Previous literature has suggested a threshold of 10 Ω for impedance drop to minimize the risk of AF recurrence.^23^ Our study dataset had around 90.4% of patients with an average impedance drop <10 Ω, suggesting that 10 Ω is an unrealistic target in actual clinical practices. Further research is needed to evaluate the threshold of impedance drop for clinical use. The Youden index provides equal weighting to the sensitivity and specificity of predicting AF recurrence. Clinically, the sensitivity may be more important than specificity in predicting recurrence if the electrophysiologist wishes to avoid AF recurrence, specifically if the safety profile of cardiac ablation is favorable. In our study dataset, only 7 patients experienced adverse events related to ablation. There was no statistically significant difference in adverse events between groups of patients with high vs low impedance drop.

### Limitations

This is an observational study using real-world data. Ascertainment of baseline clinical characteristics and outcomes relied on the billing codes and might have limited sensitivity and specificity. Additionally, comorbidity burden and outcomes might have been underestimated. The follow-up using ECG recording or Holter monitor to ascertain the AF recurrence was not available in the database. AF recurrence was defined as composite of AF hospitalization, repeat ablation, or direct current cardioversion with diagnosis of AF, or initialization of a new antiarrhythmic drug post–blanking period. This may underestimate the AF recurrence rate, especially for asymptomatic or mildly symptomatic AF recurrence. The index date of AF diagnosis was largely unknown, and chronicity of AF was undetermined. This study did not systematically evaluate all procedural data that may be associated with durable lesion creation due to the small sample size. The data available for this study provided the average change in impedance during the ablation procedure but not the relative impedance drop during the baseline impedance measure. The change in impedance during the ablation procedure without accounting for the patient’s baseline impedance may limit the prediction obtained from impedance drop (ie, relative impedance drop may be more predictive than absolute impedance drops, which is what was used in this study). Further analyses are needed to confirm the impedance drop threshold to predict AF recurrence, compare predictions using relative vs absolute impedance drop, assess the relationship between degree of impedance drop and adverse events, and generalize the results to other clinical sites and software.

## Conclusion

Local impedance drops at the right and left WACA ablations are a strong predictor of 1-year AF recurrence. A threshold of 7.2 Ω best predicted AF recurrence with the sensitivity of 0.73. It is feasible to link cardiac ablation digital data and patient’s EHR data to assess how procedural parameters predict patient outcome at a population level using an epidemiological approach.
