# Supplementary material for: Predictive utility of the impedance drop on AF recurrence using digital intraprocedural data linked to electronic health record data
Source: Heart Rhythm O2. 2024 Feb 2;5(3):174–81. doi: 10.1016/j.hroo.2024.01.006 (PMC10980921; doi:10.1016/j.hroo.2024.01.006)
Supplement: Appendix [file mmc1.docx]

Table A1. Odds ratio and 95% confidence intervals of multivariable analysis for average WACA impedance drop.

|  | Composite | AF hospitalization | Direct cardioversion |
| --- | --- | --- | --- |
| Gender |  |  |  |
| Male | Reference | Reference | Reference |
| Female | 0.46 (0.15, 1.37) | 0.22 (0.02, 1.81) | 0.47 (0.14, 1.49) |
| AF type |  |  |  |
| Paroxysmal AF | Reference | Reference | Reference |
| Persistent AF | 3.97 (1.60, 10.5) | 6.59 (1.13, 50.1) | 3.19 (1.21, 9.08) |
| CHA_2_DS_2_VASC score |  |  |  |
| 0 | Reference | Reference | Reference |
| 1-2 | 2.06 (0.48, 10.2) | 0.3 (0.01, 5.87) | 1.32 (0.28, 7.36) |
| >=3 | 3.65 (0.58, 25.8) | 0.3 (0.01, 13.5) | 2.12 (0.29, 17.2) |
| Elixhauser comorbidity score |  |  |  |
| 0 | Reference | Reference | Reference |
| 1-2 | 1.55 (0.31, 8.55) | 0.69 (0.03, 25.7) | 0.97 (0.17, 6.61) |
| >=3 | 1.19 (0.21, 7.23) | 2.28 (0.09, 98.8) | 0.88 (0.13, 6.62) |
| Average WACA impedance drop |  |  |  |
| (8,12.5] | Reference | Reference | Reference |
| (6.45,8] | 1.8 (0.57, 5.98) | 0.37 (0.03, 4.32) | 1.37 (0.39, 4.98) |
| [3.2,6.45] | 5.91 (1.81, 21.8) | 1.99 (0.21, 29.8) | 5.53 (1.56, 22.4) |
| Left ventricular ejection fraction |  |  |  |
| Normal ejection function | Reference | Reference | Reference |
| Missing | 1.43 (0.42, 4.77) | 0.49 (0.03, 4.56) | 0.8 (0.20, 2.96) |
| Reduced ejection function | 0.80 (0.20, 2.91) | 0.57 (0.02, 7.38) | 0.67 (0.14, 2.65) |
| Age | 1.00 (0.95, 1.06) | 1.13 (0.99, 1.31) | 1.01 (0.95, 1.07) |
| Use of antiarrhythmic drug | 0.37 (0.13, 1.04) | 3.84 (0.45, 63.7) | 0.40 (0.14, 1.19) |
| Sleep apnea | 1.06 (0.44, 2.53) | 0.05 (0.00, 0.38) | 1.20 (0.48, 3.01) |
| Average WACA stability | 0.69 (0.25, 1.90) | 0.98 (0.13, 7.42) | 0.75 (0.24, 2.32) |
| Average WACA ablation index | 0.99 (0.98, 1.01) | 0.98 (0.95, 1.02) | 0.99 (0.97, 1.01) |
| Average WACA contact force | 0.93 (0.81, 1.07) | 0.96 (0.72, 1.22) | 0.93 (0.78, 1.08) |
| Average WACA power | 1.04 (0.91, 1.16) | 1.33 (1.05, 1.76) | 0.97 (0.83, 1.10) |
| Carina | 0.79 (0.29, 2.19) | 0.94 (0.12, 8.57) | 0.62 (0.21, 1.84) |
| Ablation line outside of right/left PV | 0.71 (0.21, 2.53) | 0.18 (0.02, 1.47) | 1.72 (0.45, 8.59) |
| Dilation | 3.69 (1.29, 11.2) | 4.51 (0.61, 42.1) | 2.77 (0.92, 8.61) |
| Total ablation sites | 0.99 (0.98, 1.00) | 0.97 (0.93, 1.01) | 0.99 (0.97, 1.00) |
| Ablation duration | 1.05 (1.00, 1.11) | 1.09 (0.96, 1.24) | 1.06 (1.00, 1.12) |

Notes: WACA: wide antral circumferential ablation; AF: Atrial fibrillation; Composite outcome includes AF hospitalization, repeat ablation, DCCV, and initialization of new AAD drugs post blanking period.

Table A2. Odds ratio and 95% confidence intervals of multivariable analysis for right WACA impedance drop.

|  | Composite | AF hospitalization | Direct cardioversion |
| --- | --- | --- | --- |
| Gender |  |  |  |
| Male | Reference | Reference | Reference |
| Female | 0.46 (0.15, 1.34) | 0.3 (0.03, 2.15) | 0.56 (0.18, 1.68) |
| AF type |  |  |  |
| Paroxysmal AF | Reference | Reference | Reference |
| Persistent AF | 3.88 (1.54, 10.5) | 6.39 (1.09, 49.6) | 3.03 (1.15, 8.56) |
| Right WACA impedance drop |  |  |  |
| (8,12.5] | Reference | Reference | Reference |
| (6.45,8] | 0.44 (0.09, 1.79) | 0.25 (0.01, 4.33) | 0.58 (0.12, 2.40) |
| [3.2,6.45] | 3.80 (1.23, 13.0) | 1.50 (0.18, 17.2) | 2.68 (0.83, 9.58) |
| CHA_2_DS_2_VASC score |  |  |  |
| 0 | Reference | Reference | Reference |
| 1-2 | 2.52 (0.55, 13.4) | 0.41 (0.02, 8.17) | 1.76 (0.36, 10.1) |
| >=3 | 4.6 (0.63, 39.0) | 0.31 (0.01, 12.3) | 2.7 (0.35, 24.2) |
| Elixhauser comorbidity score |  |  |  |
| 0 | Reference | Reference | Reference |
| 1-2 | 1.77 (0.35, 10.0) | 0.89 (0.05, 28.6) | 1.15 (0.20, 7.70) |
| >=3 | 1.35 (0.22, 8.57) | 2.90 (0.13, 111) | 0.98 (0.14, 7.39) |
| Left ventricular ejection fraction |  |  |  |
| Normal ejection function | Reference | Reference | Reference |
| Missing | 1.42 (0.42, 4.75) | 0.70 (0.06, 5.62) | 0.80 (0.21, 2.85) |
| Reduced ejection function | 0.91 (0.23, 3.30) | 0.65 (0.02, 8.48) | 0.79 (0.18, 3.01) |
| Age | 1.01 (0.95, 1.07) | 1.12 (0.99, 1.30) | 1.02 (0.96, 1.08) |
| Use of antiarrhythmic drug | 0.38 (0.13, 1.10) | 4.49 (0.53, 66.1) | 0.46 (0.15, 1.36) |
| Sleep apnea | 0.94 (0.37, 2.33) | 0.05 (0.00, 0.40) | 1.11 (0.43, 2.83) |
| Average WACA stability | 0.73 (0.26, 2.02) | 0.76 (0.10, 5.36) | 0.80 (0.27, 2.38) |
| Average WACA ablation index | 0.99 (0.98, 1.01) | 0.98 (0.95, 1.02) | 0.99 (0.97, 1.01) |
| Average WACA contact force | 0.94 (0.81, 1.09) | 0.98 (0.73, 1.25) | 0.93 (0.79, 1.09) |
| Average WACA power | 1.03 (0.91, 1.16) | 1.28 (1.01, 1.70) | 0.97 (0.83, 1.10) |
| Carina | 0.85 (0.31, 2.39) | 1.08 (0.15, 9.54) | 0.7 (0.24, 2.05) |
| Ablation line outside of right/left PV | 0.74 (0.22, 2.64) | 0.21 (0.02, 1.73) | 1.69 (0.46, 8.25) |
| Dilation | 3.19 (1.08, 9.89) | 3.22 (0.40, 30.1) | 2.14 (0.71, 6.58) |
| Total ablation sites | 0.99 (0.98, 1.00) | 0.97 (0.94, 1.01) | 0.99 (0.98, 1.00) |
| Ablation duration | 1.06 (1.01, 1.12) | 1.09 (0.96, 1.24) | 1.05 (1.00, 1.11) |

Notes: WACA: wide antral circumferential ablation; AF: Atrial fibrillation; Composite outcome includes AF hospitalization, repeat ablation, DCCV, and initialization of new AAD drugs post blanking period.

Table A3. Odds ratio and 95% confidence intervals of multivariable analysis for left WACA impedance drop.

|  | Composite | AF hospitalization | Direct cardioversion |
| --- | --- | --- | --- |
| Gender |  |  |  |
| Male | Reference | Reference | Reference |
| Female | 0.49 (0.16, 1.43) | 0.35 (0.03, 2.53) | 0.53 (0.16, 1.65) |
| AF type |  |  |  |
| Paroxysmal AF | Reference | Reference | Reference |
| Persistent AF | 3.56 (1.43, 9.39) | 6.45 (1.03, 55.1) | 2.75 (1.04, 7.69) |
| Left WACA impedance drop |  |  |  |
| (8,12.5] | Reference | Reference | Reference |
| (6.45,8] | 1.50 (0.57, 3.98) | 0.31 (0.03, 2.46) | 1.68 (0.60, 4.82) |
| [3.2,6.45] | 4.48 (1.57, 13.5) | 1.57 (0.21, 13.9) | 4.34 (1.43, 14.2) |
| CHA_2_DS_2_VASC score |  |  |  |
| 0 | Reference | Reference | Reference |
| 1-2 | 1.97 (0.46, 9.60) | 0.36 (0.02, 6.20) | 1.4 (0.29, 7.82) |
| >=3 | 3.33 (0.53, 23.4) | 0.19 (0.00, 5.84) | 2.11 (0.29, 17.2) |
| Elixhauser comorbidity score |  |  |  |
| 0 | Reference | Reference | Reference |
| 1-2 | 1.02 (0.22, 5.41) | 1.03 (0.06, 33.1) | 0.72 (0.13, 4.77) |
| >=3 | 0.81 (0.15, 4.84) | 3.09 (0.14, 120) | 0.66 (0.10, 4.86) |
| Left ventricular ejection fraction |  |  |  |
| Normal ejection function | Reference | Reference | Reference |
| Missing | 1.47 (0.45, 4.78) | 0.71 (0.06, 5.57) | 0.83 (0.21, 3.01) |
| Reduced ejection function | 0.84 (0.22, 3.00) | 1.00 (0.04, 12.3) | 0.76 (0.17, 2.88) |
| Age | 1.01 (0.96, 1.07) | 1.15 (1.02, 1.34) | 1.02 (0.97, 1.08) |
| Use of antiarrhythmic drug | 0.47 (0.17, 1.26) | 5.45 (0.61, 95.0) | 0.49 (0.17, 1.42) |
| Sleep apnea | 1.19 (0.49, 2.89) | 0.06 (0.00, 0.44) | 1.31 (0.52, 3.32) |
| Average WACA stability | 0.74 (0.27, 2.02) | 0.83 (0.12, 5.49) | 0.81 (0.26, 2.44) |
| Average WACA ablation index | 0.99 (0.97, 1.00) | 0.98 (0.95, 1.01) | 0.99 (0.97, 1.01) |
| Average WACA contact force | 0.91 (0.79, 1.05) | 0.98 (0.73, 1.24) | 0.91 (0.77, 1.06) |
| Average WACA power | 1.06 (0.94, 1.19) | 1.37 (1.09, 1.82) | 0.99 (0.85, 1.12) |
| Carina | 0.83 (0.31, 2.26) | 0.69 (0.09, 5.70) | 0.67 (0.23, 1.96) |
| Ablation line outside of right/left PV | 0.79 (0.24, 2.84) | 0.26 (0.03, 2.00) | 1.82 (0.48, 9.13) |
| Dilation | 3.72 (1.30, 11.3) | 5.81 (0.69, 60.3) | 2.60 (0.86, 8.13) |
| Total ablation sites | 0.99 (0.98, 1.00) | 0.97 (0.93, 1.00) | 0.99 (0.97, 1.00) |
| Ablation duration | 1.06 (1.01, 1.12) | 1.10 (0.97, 1.26) | 1.06 (1.01, 1.13) |

Notes: WACA: wide antral circumferential ablation; AF: Atrial fibrillation; Composite outcome includes AF hospitalization, repeat ablation, DCCV, and initialization of new AAD drugs post blanking period.

Table A4. Odds ratio and 95% confidence intervals of multivariable analysis for average WACA impedance drop using threshold of 7.2 ohms.

|  | Composite | AF hospitalization | Direct cardioversion |
| --- | --- | --- | --- |
| Gender |  |  |  |
| Male | Reference | Reference | Reference |
| Female | 0.57 (0.20, 1.60) | 0.36 (0.04, 2.45) | 0.62 (0.20, 1.84) |
| AF type |  |  |  |
| Paroxysmal AF | Reference | Reference | Reference |
| Persistent AF | 3.95 (1.59, 10.4) | 6.14 (1.04, 46.7) | 3.03 (1.16, 8.47) |
| CHA_2_DS_2_VASC score |  |  |  |
| 0 | Reference | Reference | Reference |
| 1-2 | 2.29 (0.53, 11.4) | 0.34 (0.02, 5.72) | 1.68 (0.35, 9.45) |
| >=3 | 3.66 (0.57, 26.1) | 0.23 (0.01, 6.84) | 2.4 (0.33, 19.7) |
| Elixhauser comorbidity score |  |  |  |
| 0 | Reference | Reference | Reference |
| 1-2 | 1.82 (0.37, 9.97) | 1.46 (0.08, 49.3) | 1.22 (0.21, 8.11) |
| >=3 | 1.3 (0.23, 7.90) | 4.58 (0.22, 181) | 0.98 (0.15, 7.31) |
| Average impedance drop |  |  |  |
| > 7.2 | Reference | Reference | Reference |
| ≤ 7.2 | 3.51 (1.39, 9.50) | 0.94 (0.17, 5.52) | 3.03 (1.13, 8.74) |
| Left ventricular ejection fraction |  |  |  |
| Normal ejection function | Reference | Reference | Reference |
| Missing | 1.55 (0.48, 5.01) | 0.76 (0.08, 5.41) | 0.87 (0.23, 3.05) |
| Reduced ejection function | 0.89 (0.23, 3.14) | 0.88 (0.03, 10.4) | 0.78 (0.17, 2.94) |
| Age | 1.01 (0.96, 1.07) | 1.14 (1.01, 1.33) | 1.02 (0.96, 1.08) |
| Use of antiarrhythmic drug | 0.43 (0.15, 1.18) | 4.18 (0.54, 57.9) | 0.48 (0.16, 1.40) |
| Sleep apnea | 1.06 (0.45, 2.51) | 0.06 (0.00, 0.45) | 1.2 (0.48, 2.96) |
| Average WACA stability | 0.7 (0.26, 1.89) | 0.92 (0.13, 6.09) | 0.76 (0.26, 2.24) |
| Average WACA ablation index | 0.99 (0.98, 1.01) | 0.98 (0.95, 1.02) | 0.99 (0.98, 1.01) |
| Average WACA contact force | 0.92 (0.80, 1.06) | 0.95 (0.71, 1.21) | 0.92 (0.78, 1.07) |
| Average WACA power | 1.03 (0.91, 1.15) | 1.3 (1.04, 1.71) | 0.97 (0.84, 1.09) |
| Carina | 0.89 (0.34, 2.42) | 1.15 (0.17, 9.00) | 0.71 (0.25, 2.05) |
| Ablation line outside of right/left PV | 0.76 (0.24, 2.63) | 0.23 (0.03, 1.75) | 1.67 (0.46, 8.04) |
| Dilation | 3.52 (1.23, 10.6) | 3.42 (0.48, 29.4) | 2.33 (0.78, 7.13) |
| Total ablation sites | 0.99 (0.98, 1.01) | 0.97 (0.94, 1.01) | 0.99 (0.98, 1.00) |
| Ablation duration | 1.05 (1.00, 1.10) | 1.09 (0.96, 1.24) | 1.05 (1.00, 1.11) |

Notes: WACA: wide antral circumferential ablation; AF: Atrial fibrillation; Composite outcome includes AF hospitalization, repeat ablation, DCCV, and initialization of new AAD drugs post blanking period.

Table A5. Odds ratio and 95% confidence intervals of multivariable analysis for right WACA impedance drop using threshold of 7.2 ohms.

|  | Composite | AF hospitalization | Direct cardioversion |
| --- | --- | --- | --- |
| Gender |  |  |  |
| Male | Reference | Reference | Reference |
| Female | 0.63 (0.22, 1.77) | 0.4 (0.04, 2.77) | 0.69 (0.23, 2.01) |
| AF type |  |  |  |
| Paroxysmal AF | Reference | Reference | Reference |
| Persistent AF | 4.19 (1.70, 11.0) | 6.1 (1.02, 48.7) | 3.22 (1.24, 8.90) |
| Right WACA impedance drop |  |  |  |
| > 7.2 | Reference | Reference | Reference |
| ≤ 7.2 | 4.32 (1.57, 13.2) | 3.61 (0.53, 39.6) | 3.21 (1.12, 10.3) |
| CHA_2_DS_2_VASC score |  |  |  |
| 0 | Reference | Reference | Reference |
| 1-2 | 1.91 (0.44, 9.48) | 0.28 (0.02, 4.50) | 1.46 (0.31, 8.11) |
| >=3 | 3.03 (0.47, 21.4) | 0.18 (0.00, 5.51) | 2.06 (0.29, 16.6) |
| Elixhauser comorbidity score |  |  |  |
| 0 | Reference | Reference | Reference |
| 1-2 | 1.87 (0.38, 10.2) | 1.36 (0.08, 43.7) | 1.22 (0.22, 8.06) |
| >=3 | 1.46 (0.26, 8.77) | 4.53 (0.24, 165) | 1.07 (0.16, 7.86) |
| Left ventricular ejection fraction |  |  |  |
| Normal ejection function | Reference | Reference | Reference |
| Missing | 1.69 (0.53, 5.42) | 0.76 (0.08, 5.43) | 0.96 (0.26, 3.32) |
| Reduced ejection function | 1.01 (0.26, 3.65) | 0.8 (0.03, 9.89) | 0.86 (0.19, 3.30) |
| Age | 1.02 (0.96, 1.07) | 1.13 (1.00, 1.31) | 1.02 (0.97, 1.08) |
| Use of antiarrhythmic drug | 0.42 (0.15, 1.18) | 4.04 (0.52, 51.9) | 0.48 (0.16, 1.42) |
| Sleep apnea | 1.09 (0.46, 2.58) | 0.07 (0.00, 0.47) | 1.22 (0.49, 3.05) |
| Average WACA stability | 0.56 (0.20, 1.52) | 0.59 (0.07, 4.27) | 0.65 (0.22, 1.92) |
| Average WACA ablation index | 0.99 (0.98, 1.01) | 0.99 (0.95, 1.02) | 0.99 (0.98, 1.01) |
| Average WACA contact force | 0.95 (0.82, 1.09) | 1 (0.74, 1.29) | 0.94 (0.79, 1.09) |
| Average WACA power | 1.02 (0.90, 1.14) | 1.24 (0.99, 1.64) | 0.96 (0.83, 1.09) |
| Carina | 0.91 (0.34, 2.50) | 0.86 (0.12, 6.96) | 0.75 (0.26, 2.18) |
| Ablation line outside of right/left PV | 0.81 (0.26, 2.78) | 0.24 (0.03, 1.91) | 1.74 (0.48, 8.38) |
| Dilation | 3.67 (1.27, 11.1) | 5.01 (0.63, 50.3) | 2.4 (0.80, 7.40) |
| Total ablation sites | 0.99 (0.98, 1.01) | 0.98 (0.94, 1.01) | 0.99 (0.98, 1.00) |
| Ablation duration | 1.05 (1.00, 1.10) | 1.07 (0.95, 1.22) | 1.05 (1.00, 1.11) |

Notes: WACA: wide antral circumferential ablation; AF: Atrial fibrillation; Composite outcome includes AF hospitalization, repeat ablation, DCCV, and initialization of new AAD drugs post blanking period.

Table A6. Odds ratio and 95% confidence intervals of multivariable analysis for left WACA impedance drop using threshold of 7.2 ohms.

|  | Composite | AF hospitalization | Direct cardioversion |
| --- | --- | --- | --- |
| Gender |  |  |  |
| Male | Reference | Reference | Reference |
| Female | 0.56 (0.19, 1.61) | 0.34 (0.03, 2.42) | 0.56 (0.17, 1.73) |
| AF type |  |  |  |
| Paroxysmal AF | Reference | Reference | Reference |
| Persistent AF | 4.17 (1.68, 11.0) | 6.15 (1.05, 46.6) | 3.23 (1.23, 9.11) |
| Left WACA impedance drop |  |  |  |
| > 7.2 | Reference | Reference | Reference |
| ≤ 7.2 | 2.97 (1.27, 7.27) | 1.23 (0.23, 7.62) | 4.37 (1.71, 12.1) |
| CHA_2_DS_2_VASC score |  |  |  |
| 0 | Reference | Reference | Reference |
| 1-2 | 2.06 (0.48, 9.92) | 0.32 (0.02, 5.48) | 1.33 (0.28, 7.43) |
| >=3 | 3.44 (0.55, 23.7) | 0.23 (0.01, 6.68) | 2.06 (0.28, 16.8) |
| Elixhauser comorbidity score |  |  |  |
| 0 | Reference | Reference | Reference |
| 1-2 | 1.27 (0.27, 6.64) | 1.45 (0.08, 48.9) | 0.81 (0.15, 5.09) |
| >=3 | 0.93 (0.17, 5.50) | 4.26 (0.19, 173) | 0.66 (0.10, 4.78) |
| Left ventricular ejection fraction |  |  |  |
| Normal ejection function | Reference | Reference | Reference |
| Missing | 1.4 (0.43, 4.53) | 0.73 (0.07, 5.34) | 0.76 (0.19, 2.78) |
| Reduced ejection function | 0.76 (0.19, 2.70) | 0.85 (0.03, 10.2) | 0.63 (0.13, 2.46) |
| Age | 1.01 (0.96, 1.06) | 1.14 (1.01, 1.32) | 1.01 (0.96, 1.07) |
| Use of antiarrhythmic drug | 0.48 (0.17, 1.30) | 3.92 (0.51, 55.2) | 0.49 (0.16, 1.43) |
| Sleep apnea | 1.07 (0.45, 2.54) | 0.07 (0.00, 0.45) | 1.21 (0.48, 3.04) |
| Average WACA stability | 0.69 (0.25, 1.87) | 0.89 (0.13, 5.81) | 0.73 (0.24, 2.22) |
| Average WACA ablation index | 0.99 (0.98, 1.01) | 0.98 (0.95, 1.02) | 0.99 (0.97, 1.01) |
| Average WACA contact force | 0.9 (0.78, 1.03) | 0.95 (0.71, 1.21) | 0.9 (0.76, 1.05) |
| Average WACA power | 1.05 (0.93, 1.17) | 1.29 (1.04, 1.69) | 0.98 (0.84, 1.11) |
| Carina | 0.83 (0.31, 2.27) | 1.09 (0.17, 8.37) | 0.6 (0.20, 1.78) |
| Ablation line outside of right/left PV | 0.78 (0.24, 2.73) | 0.23 (0.03, 1.79) | 1.89 (0.50, 9.33) |
| Dilation | 3.43 (1.23, 10.2) | 3.65 (0.49, 31.8) | 2.75 (0.91, 8.63) |
| Total ablation sites | 0.99 (0.98, 1.00) | 0.97 (0.94, 1.01) | 0.99 (0.97, 1.00) |
| Ablation duration | 1.06 (1.01, 1.11) | 1.09 (0.96, 1.23) | 1.06 (1.01, 1.12) |

Notes: WACA: wide antral circumferential ablation; AF: Atrial fibrillation; Composite outcome includes AF hospitalization, repeat ablation, DCCV, and initialization of new AAD drugs post blanking period.


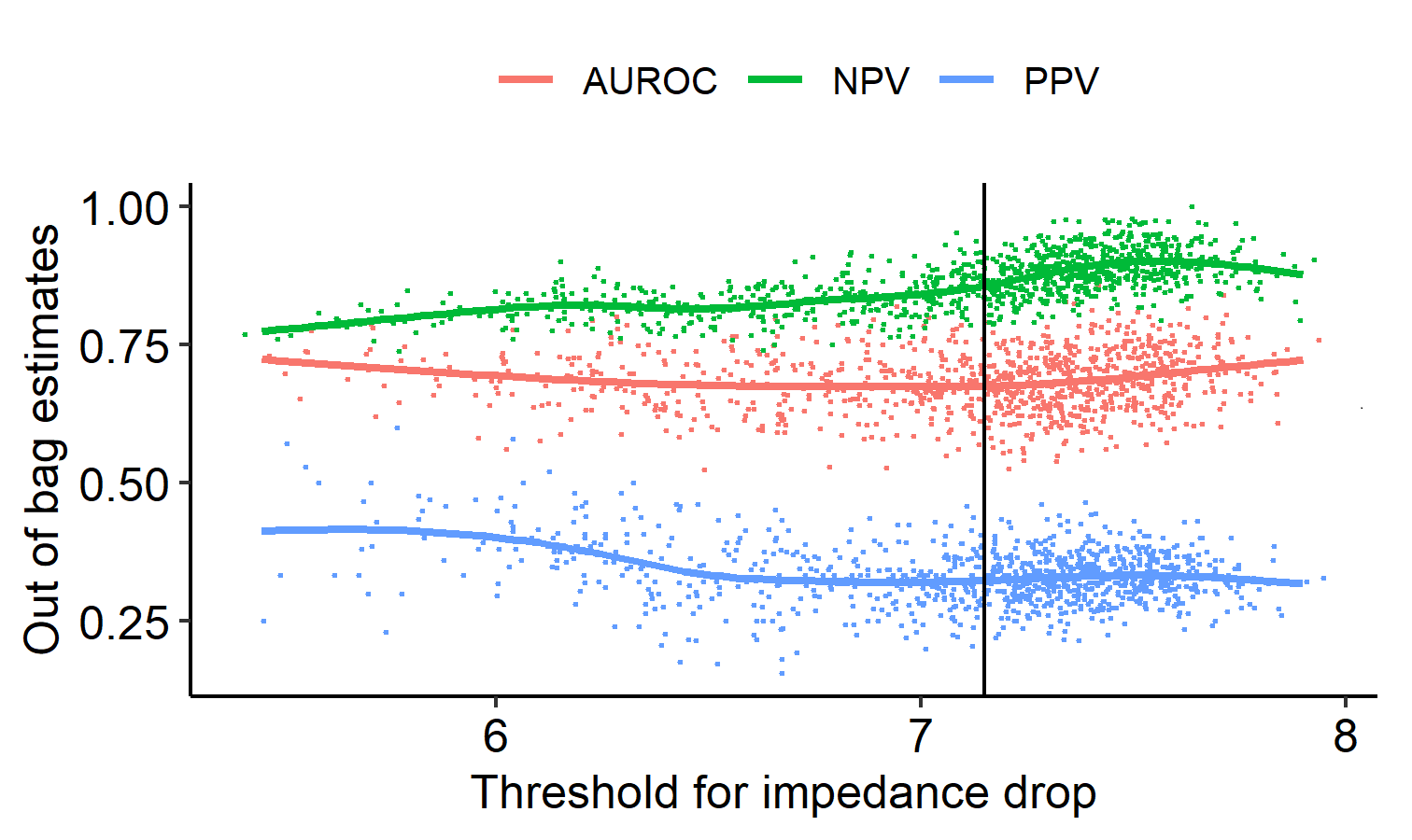


Figure A1. Results from bootstrap analysis: association of area under receiver operating curve (AUROC), positive predictive value (PPV), and negative predictive value (NPV) and impedance drop threshold values.
